# Investigation of type I interferon responses in ANCA-associated vasculitis

**DOI:** 10.1038/s41598-021-87760-4

**Published:** 2021-04-15

**Authors:** Isabella Batten, Mark W. Robinson, Arthur White, Cathal Walsh, Barbara Fazekas, Jason Wyse, Antonia Buettner, Suzanne D’Arcy, Emily Greenan, Conor C. Murphy, Zoe Wigston, Joan Ní Gabhann-Dromgoole, Edward M. Vital, Mark A. Little, Nollaig M. Bourke

**Affiliations:** 1 Department of Medical Gerontology, School of Medicine, Trinity Translational Medicine Institute, Trinity College Dublin, Dublin, Ireland; 2 Department of Biology, Kathleen Lonsdale Institute for Human Health Research, Maynooth University, Kildare, Ireland; 3 School of Computer Science and Statistics, Trinity College Dublin, Dublin, Ireland; 4 Department of Mathematics and Statistics, University of Limerick, Limerick, Ireland; 5 Regenerative Medicine Institute (REMEDI), School of Medicine, National University of Ireland Galway, Galway, Ireland; 6 Trinity Health Kidney Centre, Trinity Translational Medicine Institute, Trinity College Dublin, Dublin, Ireland; 7 Department of Ophthalmology, Royal College of Surgeons in Ireland, Dublin 2, Ireland; 8 Department of Ophthalmology, Royal Victoria Eye and Ear Hospital, Dublin 2, Ireland; 9 Leeds Institute of Rheumatic and Musculoskeletal Medicine, University of Leeds, Leeds, UK; 10 NIHR Leeds Biomedical Research Centre, Leeds Teaching Hospitals NHS Trust, Leeds, UK; 11 School of Pharmacy and Biomolecular Sciences (PBS) and RSCI Research Institute, Royal College of Surgeons in Ireland, Dublin 2, Ireland

**Keywords:** Biomarkers, Kidney diseases, Immunopathogenesis, Immunology, Innate immunity

## Abstract

Type I interferon (IFN) dysregulation is a major contributory factor in the development of several autoimmune diseases, termed type I interferonopathies, and is thought to be the pathogenic link with chronic inflammation in these conditions. Anti-neutrophil cytoplasmic antibody (ANCA)-Associated Vasculitis (AAV) is an autoimmune disease characterised by necrotising inflammation of small blood vessels. The underlying biology of AAV is not well understood, however several studies have noted abnormalities in type I IFN responses. We hypothesised that type I IFN responses are systemically dysregulated in AAV, consistent with features of a type I interferonopathy. To investigate this, we measured the expression of seven interferon regulated genes (IRGs) (*ISG15, SIGLEC1, STAT1, RSAD2, IFI27, IFI44L* and *IFIT1*) in peripheral blood samples, as well as three type I IFN regulated proteins (CXCL10, MCP-1 and CCL19) in serum samples from AAV patients, healthy controls and disease controls. We found no difference in type I IFN regulated gene or protein expression between AAV patients and healthy controls. Furthermore, IRG and IFN regulated protein expression did not correlate with clinical measurements of disease activity in AAV patients. Thus, we conclude that systemic type I IFN responses are not key drivers of AAV pathogenesis and AAV should not be considered a type I interferonopathy.

## Introduction

In recent decades, a role for altered type I interferon (IFN) responses in the development of autoimmunity has emerged^1,2^. Type I IFNs are essential mediators of immune functions and are noted for their anti-viral, anti-proliferative and immuno-regulatory properties^3^. These cytokines regulate a wide range of biological processes and, under normal homeostatic conditions, their activation is tightly regulated^4^. However, dysregulation of type I IFN responses can result in the development of inflammatory autoimmune diseases^5^. First proposed as an independent classification of autoimmune disease in 2011, such type I interferonopathies now include systemic lupus erythematosus (SLE), Aicardi Goutier syndrome, dermatomyositis and primary Sjogren’s syndrome (pSS)^1,2,6,7^.

Anti-neutrophil cytoplasmic antibody (ANCA)-associated vasculitis (AAV) is a group of systemic autoimmune disorders characterised by severe inflammation of the small blood vessels and surrounding organs^8^. Three forms of AAV are recognised, each of which are associated with different clinical presentations: microscopic polyangiitis (MPA), granulomatosis with polyangiitis (GPA) and eosinophilic granulomatosis with polyangiitis (EGPA). Unusually for an autoimmune disease, AAV has a slightly higher incidence rate in males compared to females and tends to develop later in life, with an average age of onset of 62 years old^9,10^. The disease is usually characterised by the presence of autoantibodies (ANCA) directed against the neutrophil proteins myeloperoxidase (MPO) or proteinase 3 (PR3)^11–13^ and binding of these autoantibodies results in neutrophil activation which induces a rapid onset pro-inflammatory response^14^. Early diagnosis and treatment of these disorders is crucial in preventing serious organ damage and death. However, due to a lack of knowledge regarding the underlying biology of the disease, no curative measures are currently available. Treatment options generally rely on broad immunosuppression using drugs such as corticosteroids which dampen the uncontrolled inflammation. Although these treatments reduce the cell and tissue destruction created by immune dysregulation, the nonspecific nature of these treatments often result in adverse effects and poor outcomes for patients and so a need for more select and suitable alternatives is clear^15^.

Interestingly, AAV shares both clinical and immunological similarities with known type I interferonopathies such as SLE and past studies have suggested a role for type I IFNs in AAV severity^16,17^. In 2009, while studying the process of NETosis in AAV, Kessenbrock et al. found increased *MxA* expression, a typical interferon regulated gene (IRG), in the glomeruli and tubules of active AAV patients. Additionally, they noted increased IFN-α concentrations in the serum of active AAV patients in comparison to AAV remission patients and healthy controls, suggesting a role for these cytokines in AAV activity^16^. In 2017, Ishizu et al. examined the gene expression profiles of Japanese MPA patients, noting a decrease in specific IFN regulated gene expression following remission induction treatment, and concluded that these genes are good markers of therapeutic benefit^17^. However, to our knowledge, type I IFNs have never been conclusively investigated as causative factors of AAV initiation or progression and, with specific therapies designed to target various elements of type I IFN responses currently undergoing clinical trials for the treatment of type I interferonopathies, investigations into the role of these cytokines in AAV has become even more pressing^18,19^.

We thus hypothesised that systemic type I IFN responses are dysregulated in AAV. In the present study we measured commonly used markers of type I interferonopathies in AAV patients and compared their expression to disease and healthy controls to determine whether type I IFN responses are dysregulated in AAV.

## Results

### Cohort characteristics

A total of 217 participants were recruited for this study comprising healthy controls (n = 72), disease controls (n = 34), AAV active patients (n = 48), AAV remission patients (n = 35), SLE patients (n = 19) and pSS patients (n = 9). Four of these participants provided both active and remission matched samples, one DC (anti-GBM) and 3 AAV participants. This resulted in a total of 168 serum samples, 178 whole blood samples and 30 PBMC samples (Table 1).

**Table 1:** Cohort summary: All participants have been categorised as either Healthy controls (HC), Disease controls (DC), AAV patients (Active {A} or Remission{R}), Systemic Lupus Erythematosus (SLE) or primary Sjogren’s syndrome (pSS).

Cohort summary		HC	DC	AAV R	AAV A	SLE	pSS
*N*	Participants	72	34	35	48	19	9
	Serum samples (*n*)	67	31	29	41	0	0
	Whole Blood Samples (*n*)	62	29	27	41	19	0
	PBMC samples (*n*)	5	4	6	6	0	9
Age, median (range), years		51 (16–83)	60 (16–87)	62 (25–80)	65 (22–84)	44 (28–60)	57 (52–73)
Male, *n* (%)		24 (36)	20 (57)	20 (17)	24 (50)	2 (11)	1 (11)

A summary of the samples obtained as well as the median age and sex breakdown is shown. Age and sex breakdown for the HC cohort is based off of whole blood and serum donors only (n = 67) as this data was unavailable for participants involved in PBMC analysis (n = 5).

Active AAV disease is clinically defined by a Birmingham vasculitis activity score (BVAS) of ≥ 1. However, in order to exclude cases of persistent mild inflammation from our study our active cohort were selected to have a BVAS ≥ 3. AAV patients in remission were defined by a BVAS of zero. A summary of clinical measurements used to define and study our AAV participants is shown in Table 2.

**Table 2:** AAV Characteristics: AAV participants have been divided into AAV R and AAV A cohorts with a further delineation of AAV A into treated (Txt) and treatment naïve (Txt Naïve) participants.

AAV characteristics			AAV R	AAV A	
				Txt	Txt Naive
*N*	Patients		35	37	11
ANCA status, *n* (%)	Anti-MPO		19 (54)	16 (43)	9 (82)
	Anti-PR3		16 (46)	21 (57)	2 (18)
Diagnosis, *n* (%)	GPA		16 (46)	20 (54)	1 (9)
	MPA		19 (54)	17 (46)	10 (91)
BVAS, median (range)			0	14 (3–32)	14 (12–27)
CRP (mg/dL), median (IQR)			3 (10.8–9.8)	18.5 (5.3–77.8)	46 (39–112)
Creatinine (μmol/L), median (IQR)			119.5 (86.5–166.5)	136.5 (89.5–296.5)	247 (201–416)
eGFR(ml/min), median (IQR)			49.5 (32.3–34.8)	42 (15–73)	17.5 (11–23)
**Immunosuppression treatment, n (%)**					
Cyclophosphamide	0–6 months		5 (13.9)	3 (8.1)	N/A
Azathioprine	Current		14 (38.9)	4 (10.8)	N/A
MMF	Current		9 (25)	5 (13.5)	N/A
Methotrexate	Current		2 (5.6)	0	N/A
Rituximab	0–6 months		2 (5.6)	2 (5.4)	N/A
Corticosteroid	Current		24 (66.7)	30 (81.1)	N/A
Other	Current		7 (19.4)	5 (13.5)	N/A

A summary of clinical characteristics including ANCA status, diagnosis, Birmingham Vasculitis Activity Score (BVAS), C-reactive protein (CRP) cytokine levels, creatinine, estimated Glomerular Filtration Rate (eGFR) and treatment history is shown. IQR; interquartile range.

To evaluate potential effects of general kidney inflammation and systemic autoimmunity on type I IFN responses, a disease control group comprised of patients diagnosed with a spectrum of kidney diseases and/or autoimmune disorders were included in this study. These included diagnoses of anti-glomerular basement membrane (anti-GBM) disease (n = 12), chronic kidney disease (CKD; n = 7), classical polyarteritis nodosa (PAN) (n = 1), chronic pyelonephritis (n = 1), diabetic kidney disease (n = 4), IgA vasculitis (n = 4), rheumatoid vasculitis (n = 1) and rheumatoid arthritis (n = 4). Samples collected from known type I interferonopathies (SLE; n = 19 and pSS; n = 9) were also utilised as positive disease controls in this study. Further information regarding disease activity and treatment of disease controls is shown in Supplementary Tables 1 and 2.

### Peripheral IRG expression is not upregulated in AAV

Expression of specific IRGs are elevated in blood from patients with type I interferonopathies and correlate with disease severity in these conditions^2,20,21^; therefore, we investigated the expression of a panel of these IRGs (*ISG15, SIGLEC1, STAT1, RSAD2, IFI27, IFI44L* and *IFIT1*) using mRNA extracted from whole blood samples. These genes are upregulated in the presence of type I IFNs and are often adopted as surrogate markers of type I IFN activity which can otherwise be difficult to detect^4,5,18,22,23^. They are continuously found to be dysregulated in transcriptomic analysis of known type I interferonopathies and are therefore commonly used to investigate the transcriptional activity of type I IFNs in such disease settings (Supplementary Table 3)^2,6,18,20^.

**Figure 1:**
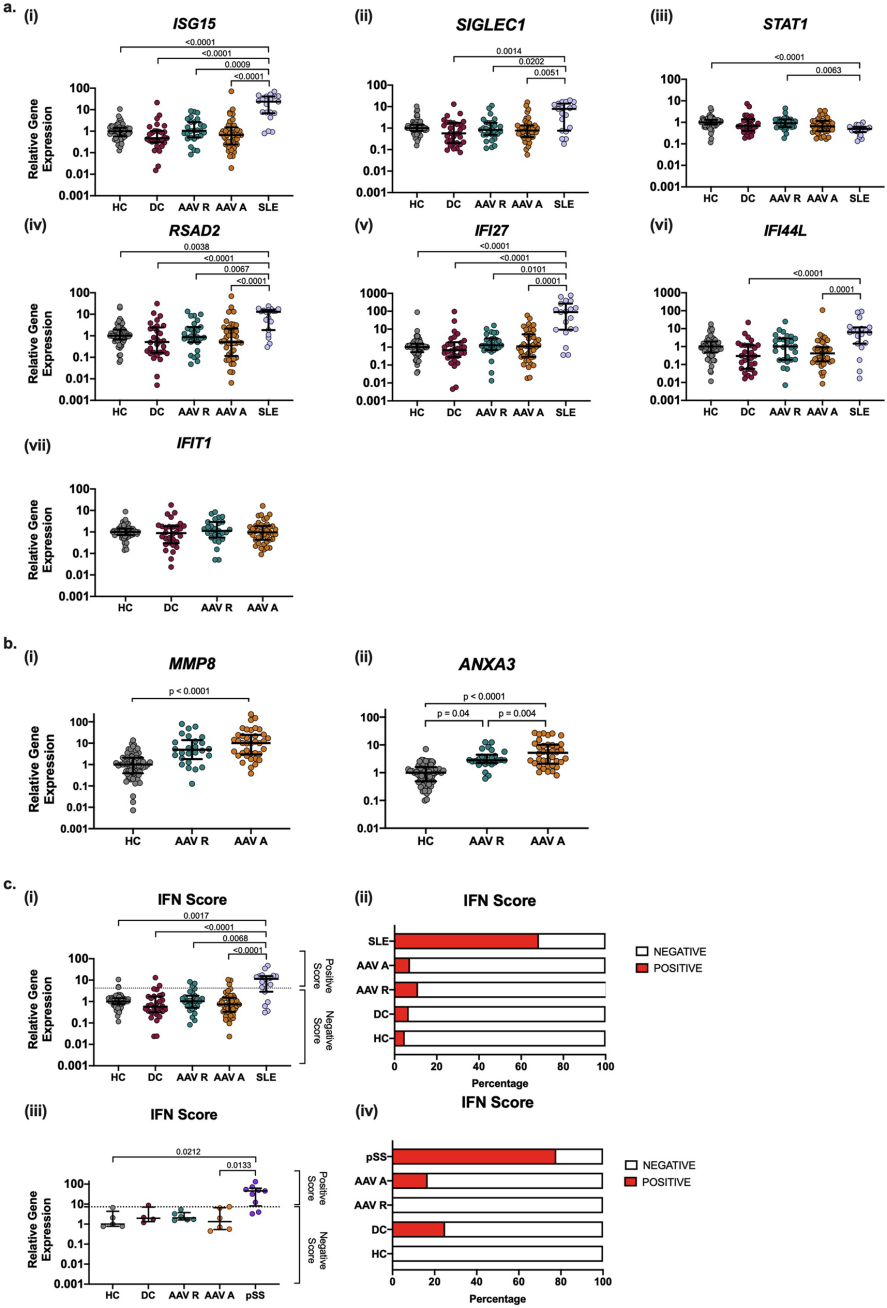
IRG expression in AAV patients and controls. Whole blood samples were obtained from healthy controls (HC; n = 62), disease controls (DC; n = 29), AAV remission patients (AAV R; n = 27), AAV active patients (AAV A; n = 41) and SLE patients (n = 19). PBMC samples were obtained from healthy controls (HC; n = 5), disease control (DC; n = 4), AAV remission (AAV R; n = 6), AAV Active (AAV A; n = 6) and primary Sjogren’s syndrome (pSS; n = 9). qPCR was used to quantify gene expression and all data is shown relative to the expression of the endogenous control gene *RPL27* and normalised to the median expression of the healthy control samples (2^−ΔΔCT^). (**a**) Gene expression measurements of the IRGs (i) *ISG15*, (ii) *SIGLEC1,* (iii) *STAT1* (iv) *RSAD2* (v) *IFI27,* and (vi) *IFI44L* in whole blood from HC, DC, AAV R, AAV A and SLE groups, (vii) *IFIT1* in HC, DC, AAV R and AAV A groups only. Due to technical issues *IFIT1* expression analysis could not be performed on SLE samples. (**b**) Gene expression measurements of (i) *MMP8* and (ii) *ANXA3* in whole blood HC, AAV R and AAV A groups. (**c**) (i) IFN scores calculated and plotted for each whole blood sample analysed. (ii) Percentage of positive (red) and negative (white) IFN scores in each cohort studied from whole blood samples. (iii.) IFN scores calculated and plotted for each PBMC sample analysed. (iv.) Percentage of positive (red) and negative (white) IFN scores in each cohort studied from PBMC samples. Whole horizontal lines represent the median and IQR of each cohort. Statistical analysis was performed using One-Way ANOVA with Dunn’s multiple comparison testing.

No significant difference in whole blood IRG expression was noted between AAV samples (active or remission), disease controls or healthy control groups, whereas samples collected from a known type I Interferonopathy, SLE, showed notably increased expression across all genes measured with the exception of *STAT1* (Fig. 1a). Expression in all samples were independent of sex while AAV data exhibited only weak to moderate correlations with age (Supplementary Figs. 1a and 2a; Supplementary Table 4).

We observed significantly higher expression of the genes *MMP8* and *AXA3* in our active AAV group compared to HCs (*p* < 0.05; Fig. 1b), neither of which are primarily type I IFN regulated^24,25^, indicating that our experimental system can detect such signals. This is consistent with existing literature^26^, confirming that our AAV cohort exhibits expected gene expression characteristics, but that systemic IRG expression is not specifically altered in this condition.

We also calculated an IFN score for each sample in our cohort. While SLE samples showed a significantly increased IFN score compared to all other cohorts, no differences in the median IFN score was noted between AAV samples, disease controls and healthy controls (Fig. 1c). Of the 24 positive IFN score samples detected, 3 were (4.8%) healthy control, 2 (6.7%) disease control, 3 (11.1%) AAV remission patient samples, 3 (7.3%) AAV active patient samples and 13 SLE patient samples (68.4%) (Fig. 1c). The percentage of healthy control and interferonopathy groups with a positive IFN score in our study is consistent with those of previous studies^2,18^.

Changes to whole blood cell composition is common in disease pathology, including in AAV, where patients often exhibit altered leukocyte cell counts^27,28^. Different cell subsets have different gene expression profiles^29^ so varying blood cell composition can affect transcriptional analysis^30,31^. To assess any potential effects that differential cell counts may have on our analysis, the absolute cell counts available for each participant were correlated with their corresponding IFN scores. Only weak to moderate correlations, none of which were significant, were found between IFN scores and total white blood cell counts, which included cell counts for neutrophils, lymphocytes, eosinophils and platelets (Supplementary Fig. 5).

Finally, we examined this IRG signature (*ISG15, SIGLEC1, STAT1 RSAD2, IFI27* and *IFI44L*) in peripheral blood mononuclear cells (PBMCs) collected from a smaller cohort of healthy controls, disease controls, AAV and primary Sjogren’s syndrome (pSS) patients. Numerous studies have reported an upregulation of IRGs in the peripheral blood and salivary glands of pSS patients, resulting in its classification as a type I interferonopathy (Supplementary Table 3)^7,32^. We observed upregulation of IRGs in PBMCs collected from pSS patients compared to all other cohorts, yet we again found no significant differences in IRG expression between AAV (active or remission) PBMC samples and both disease and healthy control groups (Supplementary Fig. 3). We calculated IFN scores for all samples, which confirmed that pSS samples had significantly increased IFN scores compared to all other cohorts while no significant differences in the median IFN score was noted between AAV samples, disease controls and healthy controls (Fig. 1c). Of the 9 positive IFN score samples detected, 1 was a disease control sample (25%), 1 was an AAV active patient samples (16.7%) and 7 were pSS patient samples (77.8%) (Fig. 1c). Together, our results confirm that there is no elevation in the IFN transcriptional signature in either PBMC or whole blood samples from AAV patients.

### Type I IFN regulated protein expression does not differ between AAV and healthy controls

**Figure 2:**
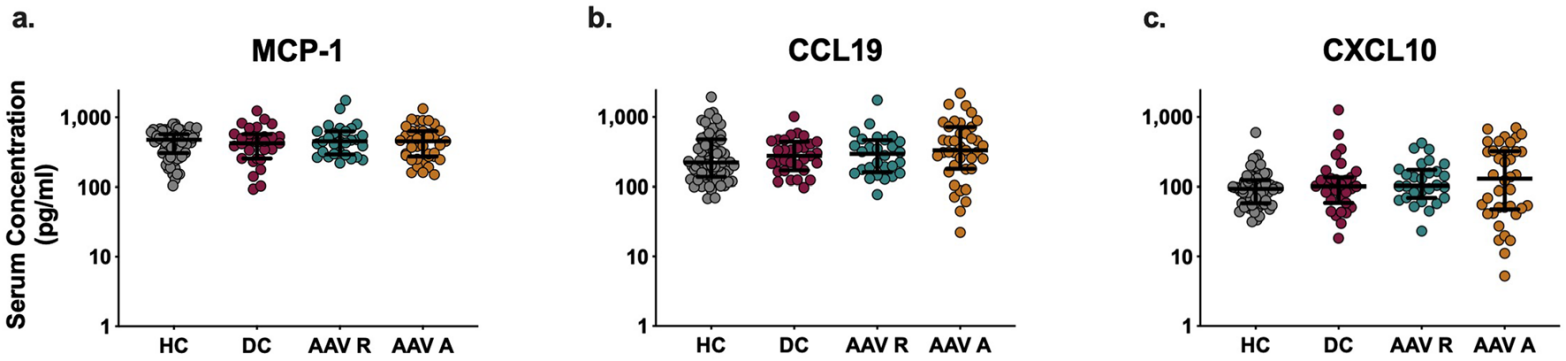
Serum concentrations of Type 1 IFN regulated chemokines expression In AAV patients and controls. Serum samples were obtained from HC (n = 67), DC (n = 31), AAV R patients (n = 29) and AAV A patients (n = 41). The concentration of circulating (**a**) MCP-1, (**b**) CCL19 and (**c**) CXCL10 were measured using ELISAs. Whole horizontal lines represent the median and IQR of each cohort. Statistical analysis was performed using One-Way ANOVA with Dunn’s multiple comparison testing. No statistical differences were observed.

We next measured the serum concentration of three type I IFN regulated chemokines: MCP-1, CCL19 and CXCL10. Although these chemokines can be regulated by several inflammatory mediators, they have previously been validated as robust biomarkers of type I interferonopathies (Supplementary Table 3)^1,33^. We found no significant differences in the serum concentration of MCP-1, CCL19 or CXCL10 in AAV patients compared to HC and DC (Fig. 2). Levels of these chemokines in all cohorts were independent of sex and showed only weak to moderate correlations with age (Supplementary Figs. 1b and 2b; Supplementary Table 4).

### Immunosuppression treatment does not affect IRG expression, but AAV treatment naïve patients have significantly increased CXCL10 serum concentrations

**Figure 3:**
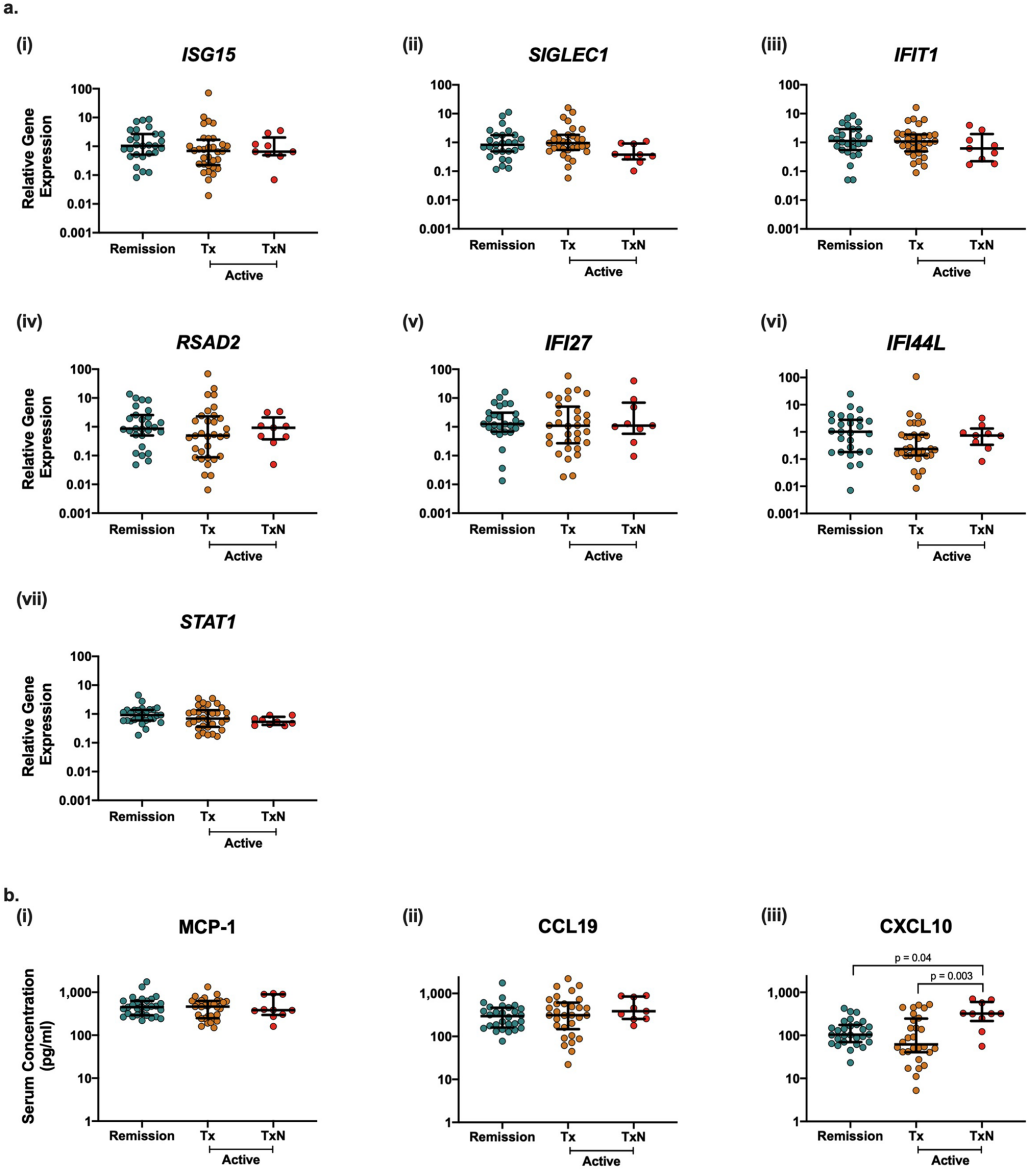
The effects of immunosuppressive treatment on type I IFN regulated gene expression and protein expression. Whole blood samples were obtained from treated AAV remission patients (n = 27), treated AAV active patients (Tx; n = 31) and treatment naïve AAV active patients (TxN; n = 10). qPCR was used to quantify the expression of seven IRGs (**a**); IRGs (i) *ISG15*, (ii) *IFIT1,* (iii) *SIGLEC1,* (iv) *RSAD2* (v) *IFI27,* (vi) *IFI44L* and (vii) *STAT1*. Gene expression data is shown relative to the expression of the endogenous control gene *RPL27* and normalised to the median expression of the healthy control samples (2^−ΔΔCT^). Matching serum samples were collected from AAV remission (n = 29), treated AAV active patients (Tx; n = 31) and treatment naïve AAV active patients (TxN; n = 10). (**b**) Serum concentrations of circulating (i) MCP-1, (ii) CCL19 and (iii) CXCL10 were measured using ELISAs. Whole horizontal lines represent the median and IQR of each cohort and statistical analysis was performed using One-Way ANOVA with Dunn’s multiple comparison testing.

The immunosuppressive treatments received by AAV patients in this cohort are known to alter the expression of inflammatory mediators. Therefore, we investigated whether treatment naïve patients had elevated expression and potential effects of these drugs on type I IFN responses in AAV. Immunosuppressive treatment did not affect IRG expression, with treatment naïve (TxN) samples and treated (Tx) samples showing similar levels for all IRGs measured (Fig. 3a).

We also observed no differences in MCP-1 or CCL19 concentrations between treated and treatment naïve samples (Fig. 3b). However, we did observe a significant increase in CXCL10 in treatment naïve samples (Fig. 3b; *p* < 0.05), which was not seen in treatment naïve disease control patients.

### IRG and type I IFN regulated protein expression does not correlate with AAV severity

To explore potential relationships between type I IFN responses and AAV severity, we examined the correlation of type I IFN regulated protein and gene expression with three clinical measurements of AAV severity: BVAS, CRP concentrations and creatinine levels. We found no significant (*p* < 0.05) or strong (r > 0.5) correlations between type I IFN regulated gene or protein expression data and any of these clinical measurements (Table 3).

**Table 3:** Correlation of type I IFN responses with clinical measurements of AAV: Whole blood IRG gene expression data, as well as type I IFN regulated protein concentration data, collected from AAV patients (both active and remission) were correlated with matched BVAS, CRP and Creatinine levels.

		BVAS	CRP	Creatinine
Gene expression	*ISG15*	− 0.18	0.04	− 0.21
	*SIGLEC1*	− 0.05	− 0.08	− 0.19
	*IFIT1*	− 0.03	− 0.06	− 0.03
	*RSAD2*	− 0.10	0.07	− 0.17
	*IFI27*	− 0.13	0.08	− 0.10
	*IFI44L*	− 0.21	− 0.12	− 0.02
	*STAT1*	− 0.12	0.08	− 0.23
Protein expression	CXCL10	0.15	0.19	0.02
	MCP-1	0.05	− 0.08	0.18
	CCL19	0.18	0.20	0.13

Spearman correlation analysis was used to generate correlation coefficient values, indicated in the table, and to determine the significance of these relationships. No statistically significant correlations were observed.

## Discussion

The purpose of this study was to investigate whether systemic type I IFN responses are dysregulated in AAV, indicative of a type I interferonopathy. Both transcriptional and translational mediators of type I IFN responses were measured following previously described methods commonly used in the identification and study of existing type I interferonopathies^20,33^. Overall, our results indicate no link between systemic type I IFN regulated gene and protein expression and AAV, suggesting that AAV is not a type I interferonopathy and that patients are therefore unlikely to benefit from interferon inhibiting therapies.

Diseases such as SLE, Aicardi Goutier syndrome, dermatomyositis and Sjogren’s syndrome are examples of autoimmune conditions that have been classified as type I interferonopathies due to alterations in systemic type I IFN responses^1,2,6,7^. Dysregulation of type I IFN responses are implicated as drivers of the meta-inflammation characteristic of these conditions and elevated type I interferon regulated gene and protein expression often act as hallmarks for these type I interferonopathies^2,20,33,34^. As well as showing abnormal IFN regulated expression, IFN activity in type I interferonopathies often directly correlates with clinical severity measurements and outcomes of these patients. In SLE, for example, IFN responses have been shown to directly correlate with the SLE disease activity index (SLEDAI)^33,35^. Despite the lack of significant differences in type I IFN regulated gene and protein expression between AAV samples and control samples we explored the idea that type I IFN responses may still have a role in AAV disease severity, postulating that high IFN regulated expression may correlate with the presence of severe acute disease. We found no strong correlations between standard clinical measurements of AAV (including BVAS, creatinine levels and CRP concentrations) and type I IFN regulated expression at either a transcriptional or a translational level. We also did not find any evidence that the cellular composition of our samples influenced the IFN scores that we generated in our AAV cohort, however we do note that we did not perform cell type specific analysis and this is a limitation of our findings. Together, our analysis suggests that type I IFNs and associated responses are not key drivers of AAV disease pathogenesis.

Treatment for AAV consists of broad immunosuppression and it is possible that immunosuppressive drugs impact on systemic type I IFN responses. We found that treatment naïve AAV patients have significantly higher concentrations of circulating CXCL10 when compared to treated patients suggesting that the immunosuppressive drugs received by these individuals act to suppress CXCL10 production. Interestingly, investigations into the role of type I IFNs in autoimmune Addison’s disease (AAD) show a similar CXCL10 finding between patients and healthy controls^36,37^. Although AAD has not officially been categorised as a type I interferonopathy, one study has found dysregulation in the negative regulators of type I IFN responses in T cells of patients with AAD following stimulation^36^. This data suggests that, at baseline, type I IFN regulated gene expression is standard in AAD but T cells isolated from these patients fail to respond appropriately to immune stimulation resulting in alterations to type I IFN regulated responses. This raises the intriguing question of whether basal type I IFN responses also remain intact in AAV but an inability to appropriately respond to immune challenges occurs, or furthermore whether dysregulation of IFN signalling is specific to certain cell types which may become diluted in whole blood analysis. It is important to note, however, that although CXCL10 has been proven to be a useful biomarker of type I interferonopathies in the past^1^, it is also known to be upregulated in several other autoimmune disorders, so this upregulation is not necessarily specific to type I interferonopathies^38^. CXCL10 expression can be upregulated by several immune factors in addition to type I IFNs, in particular IFNγ, a type II IFN. IFNγ production is reported to be upregulated in AAV and genes involved in IFNγ signalling pathways have been proposed to have novel roles in AAV pathogenesis suggesting that perhaps the upregulation of CXCL10 seen here in treatment naive AAV active patients is a result of type II IFN activity^39,40^.

Each of the proteins analysed here have been investigated in AAV previously and although our results agree with some of these studies, they contradict the findings of others. For example, similar to our observations, Monach et al. showed a significant increase of CXCL10 in AAV serum samples compared to healthy controls, however unlike our study, this find was independent of disease status while also showing no significant effects of treatment^41^. These conflicting results warrant further investigation however may be partially explained through differences in study design. For instance, samples used by Monach et al. were received through the RAVE trial, a trial testing the efficacy of rituximab in the treatment of AAV^41,42^. These patients were therefore undergoing a strict treatment regimen with little to no crossover with other medications. In contrast, participants in our treated cohort were often on various treatment options simultaneously and this has likely affected our results. Variable prednisone treatment before rituximab therapy can affect IFN scores in rheumatoid arthritis patients and so differences in treatment regimens between the studies is clearly an important variable to note^43^. Although our findings are therefore limited in that they cannot be attributed to the effects of one specific treatment, this is possibly a more realistic overview of treated AAV cohorts with combination therapies being routinely prescribed^44^. Additionally, differences in CXCL10 concentrations observed between the two studies may be a consequence of the differential assay sensitivities noted between our two methods of protein detection, with Monach et al. observing discrete but significant differences between their cohorts^41^. Indeed, the median values of circulating CXCL10 were higher in both our remission (103.6 pg/ml) and active (130 pg/ml) AAV patient samples when compared to that of healthy controls (93.47 pg/ml), however this did not reach statistical significance. It is worth noting that the median CXCL10 levels detected in our cohort (AAV, DC and HC) correspond with the serum concentration range of CXCL10 reported in the healthy control cohorts of various studies that use ELISAs as a means of protein detection^45–47^. However, it is clear that the role of CXCL10 in AAV pathogenesis remains to be fully elucidated. Again, our MCP-1 and CCL19 results both agree with^48,49^ and contradict previous studies^50,51^. However studies that contradict our findings had sample sizes significantly smaller than those used here^50,51^. Our large sample size is a strength of this work acting to minimise the likelihood of a type II error in our analysis (Supplementary Table 6).

This study is not without limitations and these are important to note. The main focus of this work was to investigate systemic type I IFN responses to determine whether AAV could be classified as a type I interferonopathy, as is standard practice in the study and diagnosis of type I interferonopathies^1,20^. As such, tissue and organ specific analysis was not investigated so a role for type I IFNs at local sites of inflammation cannot be ruled out. Several autoimmune diseases have site-specific upregulation of type I IFNs responses^52^. Indeed, increased gene expression of *MX1,* an IRG*,* was observed in the glomeruli of AAV patients with active disease by Kessenbrock et al. suggesting a localised change in IFN responses in active AAV patients^16^.

Another limitation is that only four matched active and remission samples were available for this study, three AAV participants and one in the disease control group, thus making robust analysis of matched samples difficult. Although increasing numbers of matched samples would have strengthened investigation of type I IFN responses in AAV disease progression and relapse, the stratification of our AAV cohort into active and remission groups, which even post-stratification had sufficient numbers for well-powered analysis, and the availability of matched clinical measurements provides significant insight into relationships between systemic type I IFN responses and disease activity.

The heterogenous nature of our AAV disease control cohort acts as an additional limitation to this study. Samples from a variety of diseases were purposefully chosen to analyse potential contribution of general kidney inflammation and systemic autoimmune disorders to enable the identification of AAV specific upregulation. However, for each group, small numbers, range of disease severities and treatments made singular disease comparison difficult and possibly blunted any signals that may have otherwise been observed in specific disease settings^53^.

Overall, this study was carried out with the aim of better understanding the underlying immune processes behind AAV progression with the potential to provide a more specific target for future treatment of AAV. Our work indicates that systemic type I IFN responses are not key drivers in AAV pathogenesis and so patients are unlikely to benefit from treatments that target these cytokines^18,19^. We have shown that systemic type I IFN responses are not dysregulated in AAV and that these responses do not correlate with AAV disease severity.

## Methods

### Participant recruitment

All AAV, HC and DC participant samples used in this study were provided by the Rare Kidney Disease (RKD) Registry and Biobank of Ireland (www.tcd.ie/medicine/thkc/research/rare.php) which has full ethical approval for the sampling and processing of human biological samples from the ethical committees of each participating hospital (St. Vincent’s Hospital Research ethics committee, Tallaght Hospital Research ethics committee, St. James’ Hospital Research ethics committee, and Beaumont Hospital Research ethics committee). These samples were collected and stored in accordance with the RKD sample and data collection and management protocol from participating centres across the country. SLE patient samples were obtained in from the Leeds Institute of Rheumatic and Musculoskeletal Medicine at the University of Leeds and the NIHR Leeds Biomedical Research Centre. Ethical approval for the use of these samples in such research has been provided by the South Yorkshire research ethics committee. All patients with primary Sjogren's syndrome (pSS) included in our study met the American-European Consensus classification criteria^54^ and were recruited from Royal Victory eye and Ear Hospital, Dublin. The study protocol was conducted in accordance with the Helsinki Declaration and approved by the institutional review boards of the Royal Victory eye and Ear Hospital. Every participant involved in this study has provided written informed consent.

### Gene expression analysis

Whole blood samples (HC, DC and AAV) that were previously collected and stored at − 80 °C in Qiagen PaxGene blood tubes were thawed to room temperature. RNA was extracted following the PaxGene RNA extraction kit protocol (Qiagen). These samples were bioanalysed using the Agilent RNA 6000 Nano kit on an Agilent 2100 Bioanalyzer following the procedures described in the kit guide. Samples with RNA integrity numbers (RIN) greater than 6 and yields higher than 30 ng/μl were selected for use.

Whole blood samples (SLE) that were collected in Tempus blood RNA tubes were extracted following the protocols outlined in the Preserved Blood RNA Purification Kit I (for use with Tempus Blood RNA tubes) (Norgen Biotek). These RNA samples were then subjected to DNAse treatment using the RNase-Free DNase I Kit (Norgen Biotek) in accordance with the manufacturer’s instructions.

PBMCs were isolated from whole blood using density centrifugation techniques. RNA from AAV, HC and DC PBMC samples were extracted using the PureLink RNA extraction kit (Thermo Fisher Scientific) while RNA from pSS PBMC samples were extracted using TRI reagent (Sigma) according to manufacturer’s instructions. All RNA samples extracted from PBMCs were DNAse treated using the Invitrogen DNAse I, Amplification Grade kit following the protocols provided with this kit.

Extracted RNA was immediately placed on ice, nanodropped and stored at – 80 °C. For all RNA samples extracted from whole blood 250 ng of high-quality RNA was reverse transcribed into cDNA, while for RNA samples extracted from PBMCs 500 ng of RNA was reverse transcribed into cDNA both following the protocols accompanying the ThermoFisher High-Capacity cDNA Reverse Transcription Kit. The cDNA produced was stored at − 20 °C until used.

All Primers were designed using the NCBI BLAST database to amplify a product of maximum 200 bps and were designed to be intron spanning where possible (See Supplementary Table 7). qPCR was carried out using Sybr green and ran on a QuantStudio 5 qPCR machine.

### IFN score calculation

An IFN score for each sample was calculated following previously described methods^2,18^. This was calculated as the median fold change of the seven IRGs for each sample when compared to the median of the healthy control samples. Abnormal IFN scores were defined as those higher than 2 standard deviations from the mean IFN score of healthy control samples i.e. those higher than 4.227 for whole blood gene expression analysis and 7.3 for PBMC gene expression analysis. Samples with an IFN score greater than this were labelled as IFN positive while any score below this measure were labelled as IFN negative.

### Enzyme linked immunosorbent assays (ELISAs)

ELISA was used to quantify serum concentrations of CXCL10, MCP-1 and CCL19. These were carried out following the protocols specified by each ELISA kit (BD Biosciences for CXCL10 and MCP-1; R&D Systems for CCL19).

### Data analysis and statistics

All statistical analyses were performed using GraphPad Prism v8.4.2 with the exception of the power calculations which were determined using R software. Shapiro–Wilk tests were used to determine normality. All data was found to be non-normally distributed. Therefore, statistical significance between each sample cohort was determined using Kruskal–Wallis one-way ANOVA, with Dunn’s post hoc multiple comparison tests, while Spearman’s Correlation tests were used to analyse associations between clinical measurements or demographic data and experimental data. The strength of these correlations were defined following published guidelines with Spearman correlation coefficients greater than 0.5 deemed strong, those below 0.3 deemed weak associations while those between 0.3 and 0.5 are acknowledged as moderate associations^55,56^. We confirmed the power of our analysis using a one-way balanced ANOVA power calculation as a baseline comparison. The mean number of samples per group in our experiments were used as the estimate for the per group sample size, and the conservative assumption that the standardised means of the three disease groups differed from the control group by delta = 0.25, 0.5, and 0.75 in some combination was made. Under these settings, our calculated biological power of the analyses ranged from 84 to 87% (Supplementary Table 6).

## Supplementary Information

Supplementary information

## Data Availability

All data will be made available in Excel format through the online open access repository Zenodo. 10.5281/zenodo.3949800.
