# Supplementary material for: Investigation of type I interferon responses in ANCA-associated vasculitis
Source: Sci Rep. 2021 Apr 15;11:8272. doi: 10.1038/s41598-021-87760-4 (PMC8050071; doi:10.1038/s41598-021-87760-4)
Supplement: Supplementary file 1 — Supplementary information [file 41598_2021_87760_MOESM1_ESM.docx]

**Investigation of Type I Interferon Responses in ANCA-Associated Vasculitis**

Isabella Batten^1^, Mark W. Robinson^2^, Arthur White^3^, Cathal Walsh^4^, Barbara Fazekas^5^, Jason Wyse^3^, Antonia Buettner^6^, Suzanne D’Arcy^6^, Emily Greenan^7,8^, Conor C. Murphy^7,8^, Zoe Wigston^9,10^, Joan Ní Gabhann-Dromgoole^7,11^, Edward M. Vital^9,10^, Mark A. Little^6^, *Nollaig M. Bourke^1^.

**Affiliations:**

1. Department of Medical Gerontology, School of Medicine, Trinity Translational Medicine Institute, Trinity College Dublin, Dublin, Ireland
2. Department of Biology, Kathleen Lonsdale Institute for Human Health Research, Maynooth University, Kildare, Ireland
3. School of Computer Science and Statistics, Trinity College Dublin, Dublin, Ireland
4. Department of Mathematics and Statistics, University of Limerick, Limerick, Ireland
5. Regenerative Medicine Institute (REMEDI), School of Medicine, National University of Ireland Galway, Galway, Ireland
6. Trinity Health Kidney Centre, Trinity Translational Medicine Institute, Trinity College Dublin, Ireland
7. Department of Ophthalmology, Royal College of Surgeons in Ireland, Dublin 2, Ireland.
8. Department of Ophthalmology, Royal Victoria Eye and Ear Hospital, Dublin 2, Ireland.
9. Leeds Institute of Rheumatic and Musculoskeletal Medicine, University of Leeds.
10. NIHR Leeds Biomedical Research Centre, Leeds Teaching Hospitals NHS Trust.
11. School of Pharmacy and Biomolecular Sciences (PBS) and RSCI Research Institute, Royal College of Surgeons in Ireland, Dublin 2, Ireland.

***Corresponding Author Email**: nbourke@tcd.ie

**Supplementary Table 1:**

| **Diagnosis** | **Disease Status** | | | | **TxN** | **Treatment received (>6 months)** | | | | | | | |
| --- | --- | --- | --- | --- | --- | --- | --- | --- | --- | --- | --- | --- | --- |
|  | *A* | *R* | *C* | *N/A* |  | *Cyclo* | *Aza* | *MMF* | *Mtx* | *CS* | *Anti-TNF* | *HCQ* | *O* |
| Anti-GBM | 6 | 7 | - | - | 2 | 2 | 1 | 1 | - | 6 | - | - | 2 |
| CKD | - | - | 7 | - | - | - | - | - | - | - | - | - | 3 |
| IgA V | 2 | 1 | - | 1 | 1 | - | - | 1 | - | 2 | - | - | - |
| DKD | - | - | - | 4 | - | - | - | - | - | - | - | - | 1 |
| RV | - | 1 | - | - | - | - | - | - | 1 | - | - | - | - |
| C-PAN | - | 1 | - | - | - | - | 1 | - | - | - | - | - | - |
| CP | - | - | 1 | - | - | - | - | - | - | - | - | - | 1 |
| RA | - | - | 4 | - | - | - | - | - | - | - | 4 | - | - |
| SLE | 10 | 9 | - | - | 3 | - | 3 | 5 | 2 | 5 | - | 11 | 3 |
| pSS | - | - | 9 | - |  | N/A | N/A | N/A | N/A | N/A | N/A | N/A | N/A |

**Supplementary Table 1**: Breakdown of disease control groups, systemic lupus erythematosus (SLE) and primary Sjogren’s syndrome (pSS) on the basis of disease status and treatments received. Disease control diagnoses include anti-glomerular basement membrane (anti-GBM) disease, chronic kidney disease (CKD), classical polyarteritis nodosa (C-PAN), diabetic kidney disease (DKD), IgA Vasculitis (IgA V), rheumatoid vasculitis (RV), chronic pyelonephritis (CP) and rheumatoid arthritis (RA). A; Active, R; Remission, C; chronic, N/A; not applicable, TxN; treatment naïve, Cyclo; cyclophosphamide, Aza; azathioprine, MMF; mycophenolate mofetil, Mtx; methotrexate, CS; corticosteroids, Anti-TNF; anti-tumour necrosis factor; HCQ; hydroxychloroquine, O; other.

**Supplementary Table 2:**

| **Diagnosis** | **SLE** | **pSS** |
| --- | --- | --- |
| SLEDAI, median (IQR) | 4  (2.5-6) | N/A |
| OSDI, median (IQR) | N/A | 41.2  (27.1-50) |
| ESPRI, median (IQR) | N/A | 6.7  (5-7) |
| NEI VF OP, median (IQR) | N/A | 50  (50-50) |

**Supplementary Table 2:** A summary of clinical measurement scores used to evaluate SLE or pSS severity. IQR; interquartile range.

|  | **Function** | **Type I Interferonopathies** | | | |
| --- | --- | --- | --- | --- | --- |
|  |  | SLE | AG | DM | pSS |
| *ISG15* | This gene is reported to have a various immune functions. The proteins produced by this gene can act as chemotactic factors for immune cells such as neutrophils during viral infection and have been proposed to function similarly to cytokines with several effects such as NK cell proliferation and DC maturation. As well as this the conjugated forms of these proteins mediate the process of ISGylation. This involves protein conjugation by ISG15, modifying proteins in similar manner to ubiquitination. The exact biological functions of this process remain unknown however ISGylation of viral proteins is known to inhibit viral replication [57, 58]. | [18,  20, 35, 59] | [2] |  |  |
| *Siglec1* | Codes for a protein present on myeloid cells that acts as an endocytic receptor facilitating clathrin-dependent endocytosis. Allows for the capture of viral components, facilitating the initiation of a specific targeted immune response [60, 61]. | [18,  20] | [2] |  |  |
| *RSAD2* | Known to inhibit infection/replication of a wide range of viruses including hepatitis C virus (HCV), influenza A virus, human immunodeficiency virus (HIV) etc. Exact mechanism of action remain unclear however studies on influenza A have linked this anti-viral activity to lipid raft disruption, preventing the later stages of viral replication [62-64]. | [18, 20] | [2] | [6] | [65] |
| *IFIT1* | Codes for proteins that specifically recognise viral RNA sequences, thereby recognising viral invasion and initiating an antiviral response as well as inhibiting viral RNA translation [66, 67]. | [18, 20] | [2] | [6] | [65] |
| *IFI27* | Though to have various functions in humans including type I IFN induced apoptosis and viral proteasomal degradation mediated through ubiquitination. | [18, 20] | [2] | [6] | [7, 65] |
| *IFI44L* | Upregulated in response to various viruses and is reported to inhibit the replication of HCV however mechanisms remain unclear. Also exhibits anti-tumour properties. [22, 68]. | [18, 20] | [2] | [6] | [65, 69] |
| *STAT1* | Codes for a signalling molecule involved in IFN production pathways [3, 70]. | [23] |  | [6] | [7, 65] |
| MCP-1 | An essential chemokine involved in monocyte and macrophage recruitment during inflammation [71]. | [1, 33] | [72] | [6, 20] | [73, 74] |
| CXCL10 | Chemokine that mediates immune responses through the recruitment and subsequent activation of numerous immune cells including monocytes, T cells and NK cells [38, 75]. | [1, 33] |  | [6, 20] | [69, 73] |
| CCL19 | A cytokine with various biological functions, most well studied as a chemokine responsible for the migration of T cells and mature dendritic cells during infection and inflammation [76, 77]. | [1, 33] |  | [6, 20] | [73] |

**Supplementary Table 3:**

**Supplementary Table 3: Interferon Regulated Gene and Protein Summary:** A summary of the functions of each type I interferon regulated gene and protein analysed in this study as well as references indicating their dysregulation in various type I interferonopathies (SLE; Systemic Lupus Erythematosus, AG; Aicardi Goutier Syndrome, DM; Dermatomyositis, pSS; primary Sjogren’s Syndrome)

**Supplementary Table 4:**

|  |  | **Age** | | | |
| --- | --- | --- | --- | --- | --- |
|  |  | **HC** | **DC** | **SLE** | **pSS** |
| **Gene**  **Expression** | ***ISG15*** | -0.22 | 0.02 | -0.3 | -0.56 |
|  | ***SIGLEC1*** | -0.3* | -0.02 | -0.37 | -0.56 |
|  | ***IFIT1*** | -0.17 | -0.03 | - | - |
|  | ***RSAD2*** | -0.2 | 0.02 | -0.43 | -0.03 |
|  | ***IFI27*** | -0.25* | 0.03 | -0.43 | -0.4 |
|  | ***IFI44L*** | -0.28* | 0.01 | -0.49* | -0.16 |
|  | ***STAT1*** | -0.24 | 0.04 | -0.42 | 0.41 |
| **Protein Expression** | **CXCL10** | 0.38** | 0.28 | - | - |
|  | **MCP-1** | 0.01 | 0.3 | - | - |
|  | **CCL19** | -0.08 | -0.01 | - | - |

**Supplementary Table 4: Correlation of type I IFN responses in HC, DC, SLE and pSS samples with age:** IRG gene expression data, as well as type I IFN regulated protein concentration data, collected from HC and DC were correlated with the corresponding age of each participant upon sample collection. Spearman correlation analysis was used to generate correlation coefficient values, indicated in the table, and to determine the significance of these relationships. * = p<0.05, ** = p<0.01.

**Supplementary Table 5:**

|  |  | **IFN Scores** | | | |
| --- | --- | --- | --- | --- | --- |
|  |  | | **DC** | **AAV R** | **AAV A** |
| **Cell Counts** | ***WBC*** | | 0.14 | 0.02 | -0.23 |
|  | ***Neutrophils*** | | 0.24 | 0.08 | -0.14 |
|  | ***Lymphocytes*** | | -0.2 | -0.18 | -0.24 |
|  | ***Eosinophil*** | | -0.04 | -0.02 | 0.08 |
|  | ***Platelets*** | | 0.15 | -0.09 | 0.03 |
|  | ***Other*** | | -0.37 | 0.13 | -0.04 |

**Supplementary Table 5: Correlation of type I IFN Scores from DC, AAV R and AAV A patients with leukocyte cell counts:** Type I IFN score data collected from DC, AAV R and AAV A patients were correlated with corresponding leukocyte cell counts measured for each participant upon sample collection. Leukocyte count data includes total white blood cell counts (WBC), neutrophil cell counts, lymphocyte cell counts, eosinophil cell counts and platelet cell counts. The remaining cells that make-up white blood cell compositions are represented as “other” in the above table. Spearman correlation analysis was used to generate correlation coefficient values, indicated in the table, and to determine the significance of these relationships. No significant correlations were noted.

**Supplementary Table 6:**

| **Variable** | **Power** |
| --- | --- |
| ISG15 | 0.846464223 |
| SIGLEC1 | 0.846464223 |
| IFIT1 | 0.84896502 |
| RSAD2 | 0.846464223 |
| IFI27 | 0.84896502 |
| IFI44L | 0.843928587 |
| STAT1 | 0.84896502 |
| IFN Score | 0.851431302 |
| CXCL10 | 0.856261625 |
| MCP1 | 0.867756297 |
| CCL19 | 0.858626319 |

**Supplementary Table 6: Power analysis of interferon regulated gene and protein work in whole blood and serum:** Power calculated using a one-way balanced ANOVA power calculation for each individual gene and protein analysed**.**

**Supplementary Table 7:**

| **Gene** | **Forward** | **Reverse** | **Length** |
| --- | --- | --- | --- |
| *RPL27* | GGGTGGTTGCTGCCGAA | GGTGCCATCATCAATGTTCTTCAC | 116 |
| *STAT1* | TGCGCGCAGAAAAGTTCCATT | AGACATCCTGCCACCTTGTG | 171 |
| *ISG15* | GCGAACTCATCTTTGCCAGT | AGCATCTTCACCGTCAGGTC | 91 |
| *SIGLEC1* | CTCTGCCTCTACCTCCACCT | AAACACGCCTCCTTCTCCAG | 152 |
| *IFIT1* | AGCTTACACCATTGGCTGCT | CCATTTGTACTCATGGTTGCTGT |  |
| *RSAD2* | CCCCAACCAGCGTCAACTAT | TCTTCTCCATACCAGCTTCCT | 147 |
| *IFI27* | ATCAGCAGTGACCAGTGTGG | TGGCCACAACTCCTCCAATC | 107 |
| *IFI44L* | ACCCCTAGAAACAGATATAGAACAA | GACGGCTGCATCTTTCAACC | 163 |
| *IFIT1* | GGACCCACAAGAATGTGAAAGC | TCACCATTTGTACACATCTCCACT | 85 |
| *MMP8* | AGCCAGGAGGGGTAGAGTTT | GGCTTATTTATTCTGCTGAACAGT | 149 |
| *ANXA3* | ACCGCGCTTTGGATTAGTGT | CAGCATCCACTGATGGGCTA | 123 |

**Supplementary Table 7: Forward and Reverse sequences of qPCR Primers.** All primers were designed using the NCBI Primer BLAST database. The forward and reverse sequences for each gene as well as the primer length is shown.

**Supplementary Figure 1: Sex Analysis of Type I IFN Regulated Gene and Protein Expression:**

Whole blood samples were obtained from healthy controls (HC; n=62), disease controls (DC; n=30), AAV remission patients (AAV R; n=27) and AAV active patients (AAV A; n=42). qPCR was used to quantify gene expression and all data was made relative to the expression of the endogenous control gene *RPL27* and normalised to the median expression of the healthy control samples (2^-ΔΔCT^). (a.) Gene expression measurements of seven IRGs (i) *ISG15*, (ii) *IFIT1,* (iii) *SIGLEC1,* (iv) *RSAD2* (v) *IFI27,* (vi) *IFI44L* and (vii) *STAT1* separated and analysed by sex (male; blue, female; pink). Matching serum samples were collected from HC (n=67), DC (n=32), AAV R patients (n=29) and AAV A patients (n=42). (b.) Serum concentrations of circulating (i) MCP-1, (ii) CCL19 and (iii) CXCL10 were measured using ELISAs and separated and analysed by sex (male; blue, female; pink). Whole horizontal lines represent the median and IQR of each cohort and statistical analysis was performed using One-Way ANOVA with Dunn’s multiple comparison testing. No significant differences were measured.

**Supplementary Figure 2: Correlation of type I IFN responses with age in AAV:**

Whole blood samples were obtained from AAV R (n=27) and AAV A patients (n=42). qPCR was used to quantify the expression of seven IRGs. All data was made relative to the expression of the endogenous control gene *RPL27* and normalised to the median expression of the healthy control samples (2^-ΔΔCT^). (a.) The correlation between age and the expression value measured for each of these IRGs (i) *ISG15*, (ii) *IFIT1,* (iii) *SIGLEC1,* (iv) *RSAD2* (v) *IFI27,* (vi) *IFI44L* and (vii) *STAT1* in both AAV A (orange) and AAV R patients (blue). Matching serum samples were collected from AAV R patients (n=29) and AAV A patients (n=42). ELISAs were used to measure the concentration of serum cytokines. (b.) The correlation between age and serum concentrations of circulating (i) MCP-1, (ii) CCL19 and (iii) CXCL10 in both AAV A (orange) and AAV R patients (blue). Trend lines represent either (a.) semilog non-linear regression lines or (b.) linear regression lines of best fit. Spearman correlations were used to determine correlation coefficients (R) and significance. * = p<0.05.

**Supplementary Figure 3: IRG Expression Measured From the PBMCs of AAV Patients and Controls**. PBMC samples were obtained from healthy controls (HC; n=5), disease controls (DC; n=4), AAV patients (n=12) (AAV R; blue and AAV A; orange) and primary Sjogren’s Syndrome patients (pSS; n=9). qPCR was used to quantify gene expression and all data is shown relative to the expression of the endogenous control gene *RPL27* and normalised to the median expression of the healthy control samples (2^-ΔΔCT^). (a.) Gene expression measurements of the IRGs (i) *ISG15*, (ii) *SIGLEC1,* (iii) *STAT1* (iv) *RSAD2* (v) *IFI27,* and (vi) *IFI44L* and in HC, DC, AAV and pSS groups. Whole horizontal lines represent the median and IQR of each cohort. Statistical analysis was performed using One-Way ANOVA with Dunn’s multiple comparison testing.
